# Refractive index sensor with magnified resonant signal

**DOI:** 10.1038/s41598-022-17676-0

**Published:** 2022-08-12

**Authors:** Zaky A. Zaky, Sagr Alamri, V. D. Zhaketov, Arafa H. Aly

**Affiliations:** 1 TH-PPM Group, Physics Department, Faculty of Science, Beni-Suef University, Beni-Suef, 62521 Egypt; 2 Department of Mechanical Engineering, College of Engineering, King Khalid University, Abha, 61421 Saudi Arabia; 3 Joint Institute for Nuclear Research, 6, Jolio-Curie St., Dubna, Moscow region Russia 141980

**Keywords:** Nanophotonics and plasmonics, Computational methods, Biophotonics

## Abstract

Herein, we theoretically suggest one-dimensional photonic crystal composed of polymer doped with quantum dots and porous silicon. The present simulated design is proposed as a refractive index biosensor structure based on parity-time symmetry. Under the parity-time conditions, the transmittance of the resonant peaks is magnified to be 57,843% for refractive index 1.350, 2725% for 1.390, 2117% for 1.392, 1502% for 1.395, 1011% for 1.399, and 847% for 1.401. By magnification, we can distinguish between different refractive indices. The present design can record an efficiency twice the published designs as clear in the comparison table. Results clear that the sensitivities are 635 nm/RIU and 1,000,000%/RIU. So, it can be used for a broader range of detection purposes.

## Introduction

Recently, the concept of electromagnetic wave dispersion and scattering has been dramatically developed since the advent of photonic crystals (PCs)^1–3^. PCs are periodic dielectric constants of different materials that have attracted high attention because of their unique behaviour like photonic bandgap (PBG)^4–7^. One-dimensional (1D-PCs) have been widely included in Tamm^8–13^, Fano^14^, and defect mode^12,15–19^ resonance to be used in various applications^20^. These resonant modes have limited amplitude (only from 0 to 100% intensity).

In the field of metamaterials, parity-time (PT) symmetric structures are received significant attention^21^. PT in optics is very similar to PT in quantum mechanics. In quantum mechanics, to achieve PT symmetry, the real component of potential (V) should be an even function of position x, while the imaginary component should be odd^21^. Similarly in optics, PT ‘optical potentials’ may be achieved by taking the real index as an even function of position and odd function of gain/loss components of refractive indices. Considering n(x) = n_R_(x) + i n_I_(x) is the optical potential, PT can be achieved if n_R_(x) equals n_R_(− x) and n_I_(x) equals − n_I_(− x). In the 1D-PC structure, PT can be realized by taking the materials of the unit cell under the above conditions to achieve amplification of the resonant peaks^22^.

Porous silicon (PSi) has now proven to have a lot of potential in biosensors and biomedicine^23–31^. PSi has a high surface area and abundant pore structures, which can be used as a good adsorbent^32^. PSi films are typically made by etching a crystalline p-type silicon substrate electrochemically using ethanol solutions and hydrofluoric acid from the top to down^33^. The pore sizes of PSi substrate can be tailored by controlling the doping level, etching current density, and the etching solution concentrations utilized^34^. In 2020, Zaky et al. excited Tamm resonance using 1D-PC of PSi and used it as a gas sensor with high efficiency^24^. Besides, they used 1D-PC-PSi to propose temperature sensors for high^35,36^ and low^37^ temperatures.

In this study, PT will be used to magnify the resonant peaks to be very distinguished in biosensing applications. The main advantage of this magnification behaviour is that only resonant peaks are magnified but the transmitted spectra on both sides of PBG are not affected. This makes the resonant peaks very remarkable and easy to be measured. The current work is distinct in some ways. For the first time, we proposed a sensor that can detect with multiple sensitivities (peak position sensitivity and amplitude sensitivity). Also, an optimization process will be done to develop the structure and achieve high performance.

## Basic equations and model design

**Figure 1:**
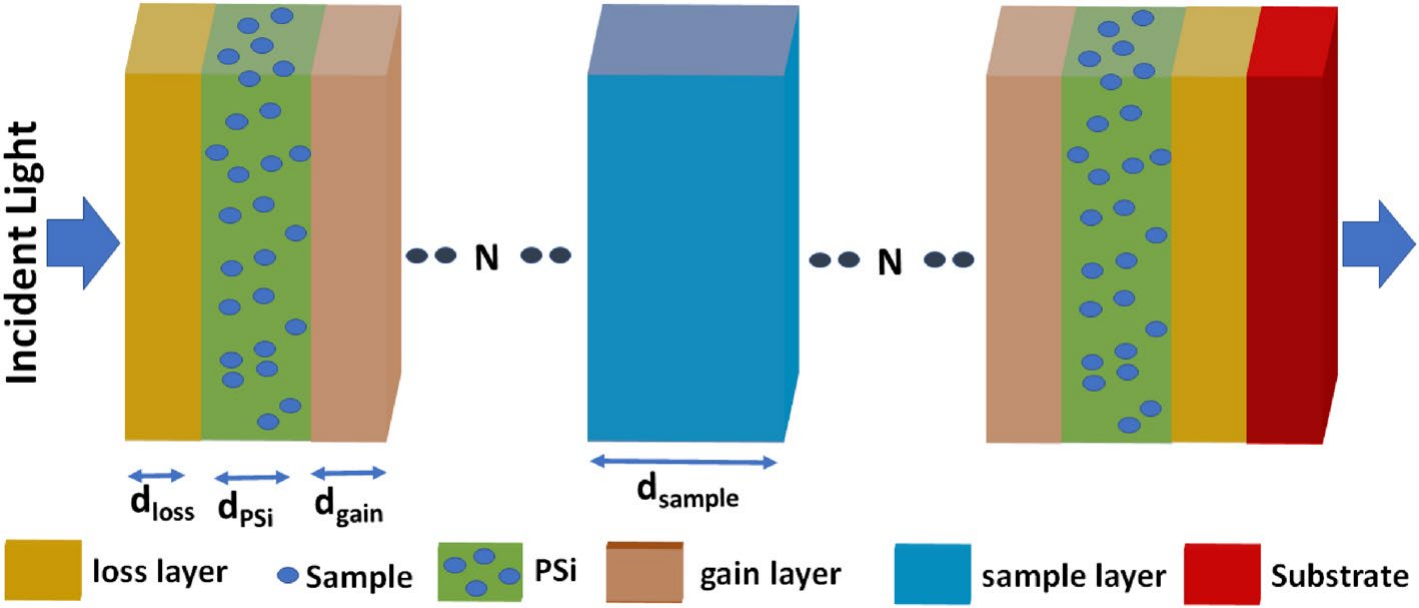
The configuration of the proposed biosensor.

Figure 1 clears a theoretically investigated configuration of (loss/PSi/gain)^N^/sample/(gain/PSi/loss)^N^/substrate as a refractive index sensor. The loss, PSi, gain, sample, and N are the loss material layer, a porous silicon layer, the gain material layer, the analyte layer that needs to be detected, and the number of unit cells of 1D-PC, respectively. The PT-symmetric criterion can be achieved by equating the thickness of loss and gain layers, satisfying the real component of the refractive indices of both loss and gain layers to the even symmetry, and the imaginary part should be odd symmetry.

The loss and the gain dielectric layers can be prepared by using a polymer substrate (${\varepsilon }_{0}=1.5$) doped with quantum dots (such as CdSe dots) using an external pump^38^. The gain layer will be excited with the external pumb, then the energy will be absorbed through the quantum dots of the gain medium by the energy-level transition (stimulated emission). At a specific wavelength, the absorbed energy will be emitted with the same energy of propagated electromagnetic waves, the coupling resonance takes place between them, and the amplification of the resonant peak is realized. Using the Lorentz model, the index of refraction of these two layers can be quantitatively calculated as^22^:

1 $${\mathrm{n}}_{\mathrm{loss }\,\&\,\mathrm{ gain}}^{ }=\sqrt{{\varepsilon }_{0}+\frac{\alpha { \omega }_{0}^{2}}{{ \omega }_{0}^{2}- {\omega }^{2}- \omega \gamma i}}$$

where $\sqrt{{\varepsilon }_{0}}$ is the refractive index of the polymer substrate of loss and gain materials, ${\omega }_{0}$ is the angular frequency at the center wavelength (${\lambda }_{0}$), $\gamma$ is the damping factor, $\omega$ is the frequency of the incident wave and $\alpha$ is the intensity of Lorentz oscillation. The refractive index of porous silicon can be calculated as^39^:

$${\mathrm{n}}_{\mathrm{Psi}}^{ }=0.5\sqrt{\uppsi +\sqrt{{\uppsi }^{2}+8 {\mathrm{n}}_{\mathrm{si}}^{2} {\mathrm{n}}_{\mathrm{sample }}^{2}}}$$

2 $$\uppsi =3\mathrm{ P }\left({\mathrm{n}}_{\mathrm{sample }}^{2}-{\mathrm{n}}_{\mathrm{si}}^{2}\right)+\left(2 {\mathrm{n}}_{\mathrm{si}}^{2}-{\mathrm{n}}_{\mathrm{sample }}^{2}\right)$$

where P is the ratio of the pores. ${\mathrm{n}}_{\mathrm{sample}}$ and ${\mathrm{n}}_{\mathrm{si}}$ are the refractive indices of silicon and the analyte sample inside the pores, respectively.

The transmittance (T) for the transverse electric (TE) polarization can be extracted by the transfer matrix method (TMM) to study the interaction between the PT-1D-PC structure with ${\theta }_{i}$ incident waves at each interface^40^:

3 $$T\left(\mathrm{\%}\right)=100*\frac{{p}_{air}}{{p}_{substrate}}{\left|t\right|}^{2},$$

where,

4 $$t=\frac{2{p}_{substrate}}{\left({A}_{11}+{A}_{12}{p}_{air}\right){p}_{substrate}+\left({A}_{21}+{A}_{22}{p}_{air}\right)},$$

5 $$\left|\begin{array}{cc}{A}_{11}& {A}_{12}\\ {A}_{21}& {A}_{22}\end{array}\right|={\left({a}_{loss}{a}_{PSi}{a}_{gain}\right)}^{N}/{a}_{sample}/{\left({a}_{gain}{a}_{PSi}{a}_{loss}\right)}^{N},$$

6 $${p}_{i}={n}_{i} cos\left({\theta }_{i}\right),\mathrm\,{ for \,TE }$$

7 $${a}_{i}=\left[\begin{array}{cc}cos{\sigma }_{i}& \left(-\frac{i}{{\mathrm{\varnothing }}_{i}}\right)sin{\sigma }_{i}\\ -i{\mathrm{\varnothing }}_{i}sin{\sigma }_{i}& cos{\sigma }_{i}\end{array}\right],$$

8 $${\sigma }_{i}=\frac{2\pi }{\lambda }{d}_{i}{n}_{i}cos{\theta }_{i} \,and\, {\mathrm{\varnothing }}_{i}= {n}_{i}cos{\theta }_{i},$$

## Results and discussions

### Sensing performance

According to Fig. 1, the 1D-PC unit cell is composed of loss and gain medium layers separated by the PSi layer with a porosity of 55% and thicknesses d_loss_ = d_gain_ = 1020 nm and d_PSi_ = 920 nm. The pores of PSi will be filled with the sample. The sample layer with thickness d_sample_ = 1500 nm is sandwiched between two asymmetrical 1D-PCs with 5 periods. The values of $\alpha$_loss_, $\alpha$_gain_, $\gamma$ and ${\omega }_{0}$ are 2.3 × 10^–4^, − 2.3 × 10^–4^, 2.5 × 10^14^ s^−1^, and 1.216 × 10^15^ s^−1^, respectively. The refractive indices of the analyte are 1.350, 1.390, 1.392, 1.395, 1.399 and 1.401.

**Figure 2:**
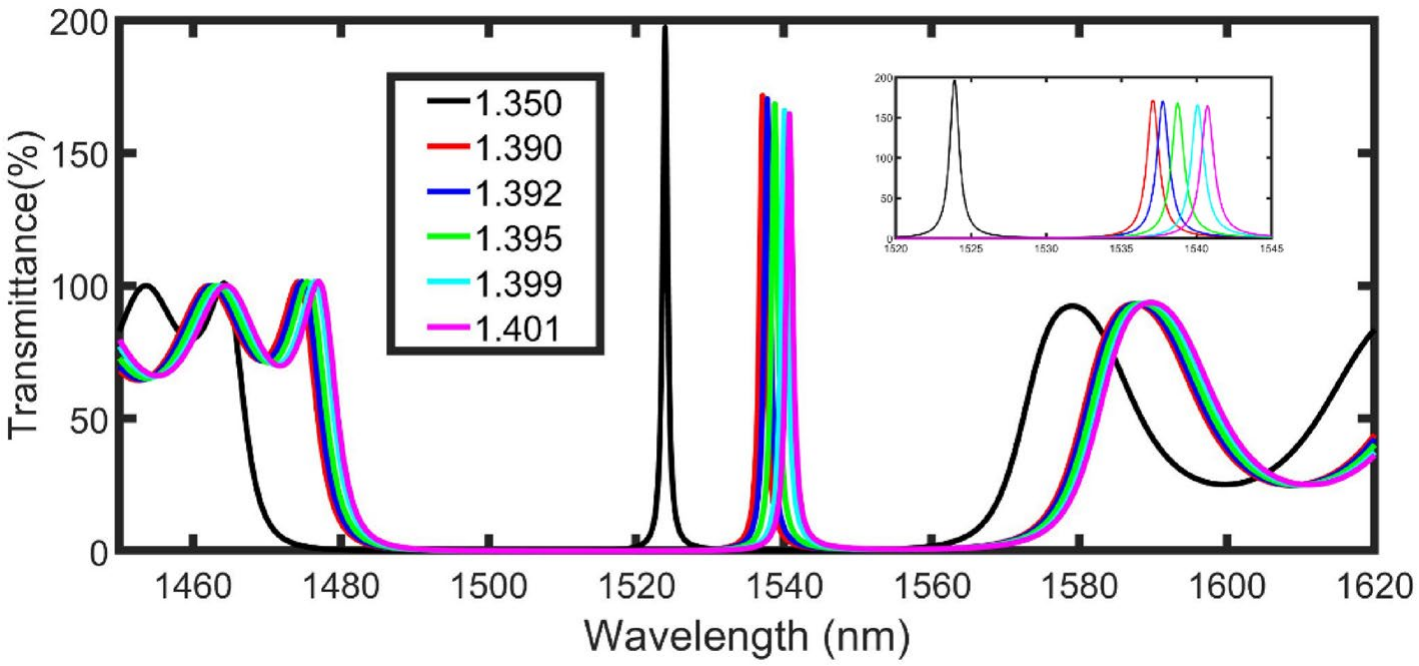
The transmittance of the proposed configuration at the suggested initial conditions with normal incidence. The inset shows a zoom of the peaks.

Figure 2 displays the transmittance of the proposed configuration at the suggested initial conditions with normal incidence. In general, due to the periodicity of the structure, PBG extended from 1475 to 1560 nm for the sample refractive index of 1.350. Besides, as a result of the defect layer and PT conditions, a magnified resonant peak appeared at 1523.9 nm with an intensity of 197%.

According to the following equation (Eq. 9) of standing wave^41^, the magnified resonant peak is shifted to high wavelengths by increasing the dielectric constant of the medium, as clear in Fig. 2(inset figure).

9 $$m\uplambda ={\mathrm{n}}_{eff} G,$$

where $\uplambda$ is the wavelength, $m$ is an integer, ${\mathrm{n}}_{eff}$ represents the square root of the effective dielectric constant of the structure, and G is the geometric path difference. By changing the n_sample_ from 1.350 to 1.390, 1.392, 1.395, 1.399, and 1.401, the magnified resonant peak is shifted from 1523.9 nm to the right side (longer wavelengths) at 1537.1, 1537.8, 1538.8, 1540.1, and 1540.7 nm, as clear in Fig. 2 (inset figure).

The sensitivity (S), figure of merit (FoM), quality factor (Q), and detection limit (LoD) will be calculated to check the performance of the model by following equations^42^:

10 $$S \left(nm/RIU\right)=\frac{\Delta {\uplambda }_{R}}{\Delta {n}_{sample}},$$

11 $$S \left(\mathrm{\%}/RIU\right)=\frac{\Delta ({\mathrm{intensity})}_{R}}{\Delta {n}_{sample}},$$

12 $$FoM=\frac{S}{FWHM},$$

13 $$\begin{array}{l}\mathrm{Q}=\frac{{\uplambda }_{\mathrm{R}}}{\mathrm{FWHM}}\end{array},$$

14 $$\mathrm{LoD}=\frac{{\uplambda }_{\mathrm{R}}}{20\mathrm{ S Q}},$$

where FWHM if the bandwidth of the magnified peak, and ${\uplambda }_{\mathrm{R}}$ is the position of it. The S (nm/RIU), S (%/RIU), FoM, Q, and LoD at the suggested initial conditions with normal incidence are 328 nm/RIU, 20,917%/RIU, 3102 RIU^−1^, 14,421, and 2 × 10^–5^, respectively. The impact of some parameters such as the number of unit cells N, sample thickness (d_sample_), and intensity of Lorentz oscillation (α) will be studied.

**Figure 3:**
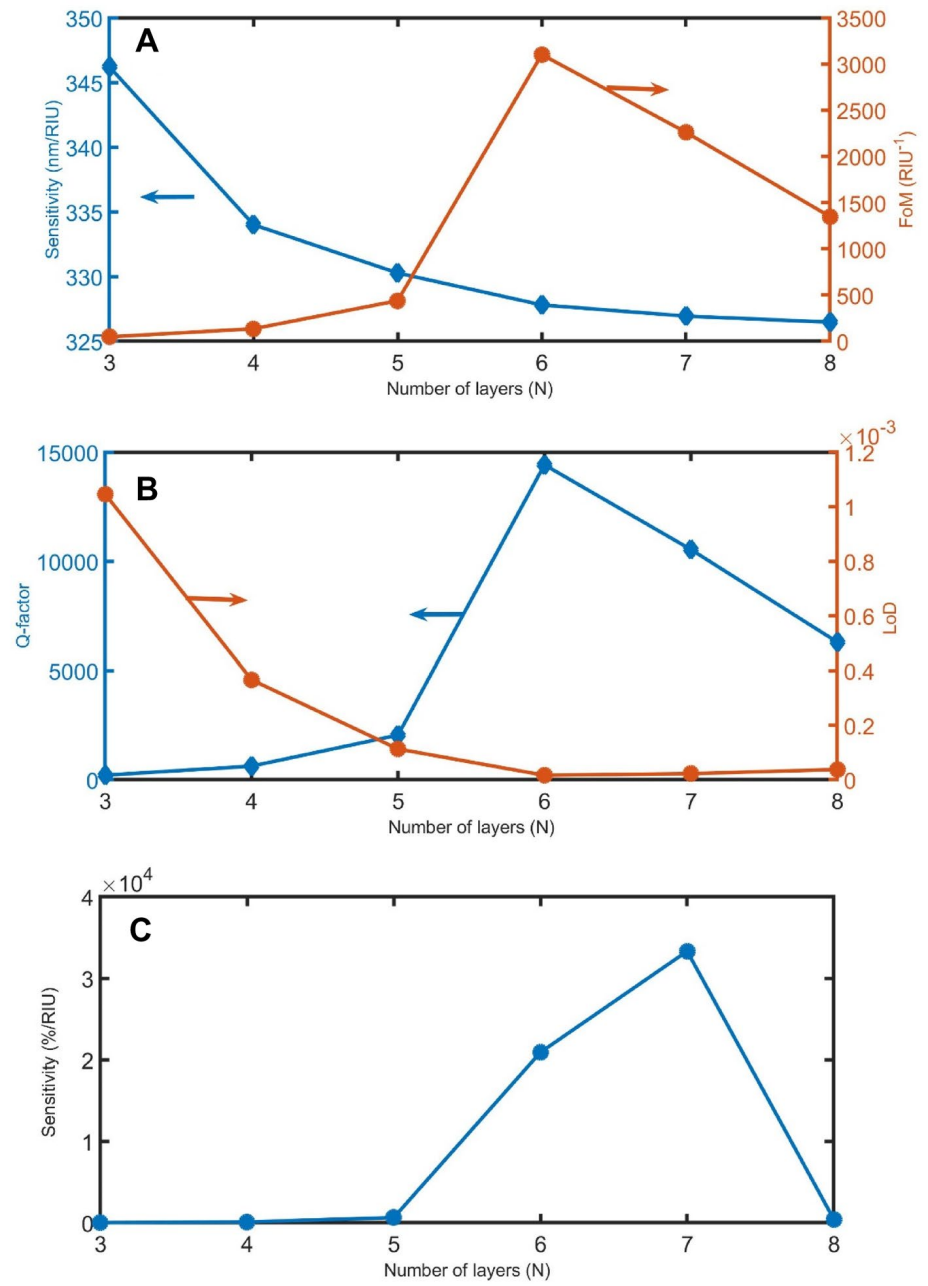
The impact of N on (**A**) sensitivity in nm/RIU and FoM, (**B**) Q and LoD, and (**C**) sensitivity in %/RIU.

Figure 3A shows the impact of N on sensitivity and FoM. With increasing of N from 3 to 4, 5, 6, 7, and 8, the sensitivity slightly decreases from 346, 343, 330, 328, 327 and 326 nm/RIU respectively. This negative effect of N is due to the increase of N causes a decrease in the volume fraction of samples in the whole structure. So, the change of n_sample_ will cause a small impact on n_eff_. For FoM, the performance of the PT-1D-PC is more satisfactory when FoM is higher as possible. Therefore, according to Fig. 3A, the FoM value at N = 6 is suitable for the optimum condition. This increase in FoM at N = 6 is due to the FWHM having the lowest value at this condition and FoM is inversely proportional to FWHM as clear in Eq. (12).

It is clear from Fig. 3B that LoD and Q exactly have the same behavior of sensitivity and FoM in Fig. 3A, respectively. This similarity between sensitivity and LoD is due to the direct proportionality between them. On the other hand, both FoM and Q are directly proportioned to FWHM.

Besides, the sensitivity in the %/RIU unit is clear in Fig. 3C. With the increase of N from 3 to 5, the difference in the magnified resonant mode intensity slightly increases from 1 to 33% by changing the n_sample_ from 1.350 to 1.401. By increasing N from 5 to 6, the difference in the magnified resonant mode intensity sharply increases to 1067%. At N = 7, the difference in the magnified resonant mode intensity recorded the maximum value of 1700%. Then, it strongly decreases to 21% at N = 8.

**Figure 4:**
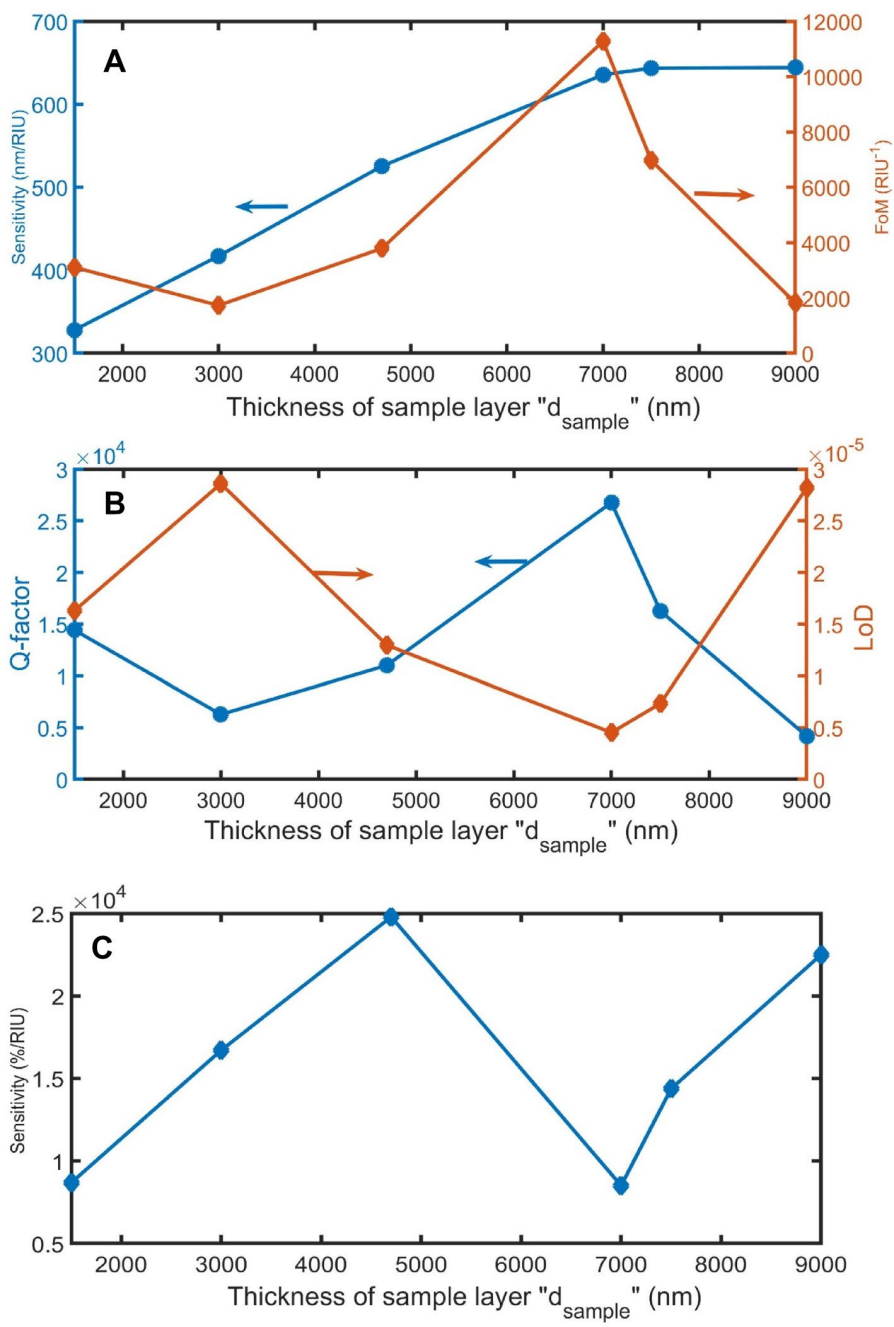
The impact of d_sample_ on (**A**) sensitivity in nm/RIU and FoM, (**B**) Q and LoD, and (**C**) sensitivity in %/RIU.

Figure 4 illustrates that the increase of d_sample_ from 1500 to 7000 nm has a strong positive effect on sensitivity in nm/RIU. After that, sensitivity slightly changes. Besides, the FoM and Q are greater at 7000 nm. And luckily, LoD becomes less valuable at this thickness. Although the thickness of 7000 nm has the lowest sensitivity in %/RIU, the thickness of 7000 nm will be favorable because of the above advantages.

**Figure 5:**
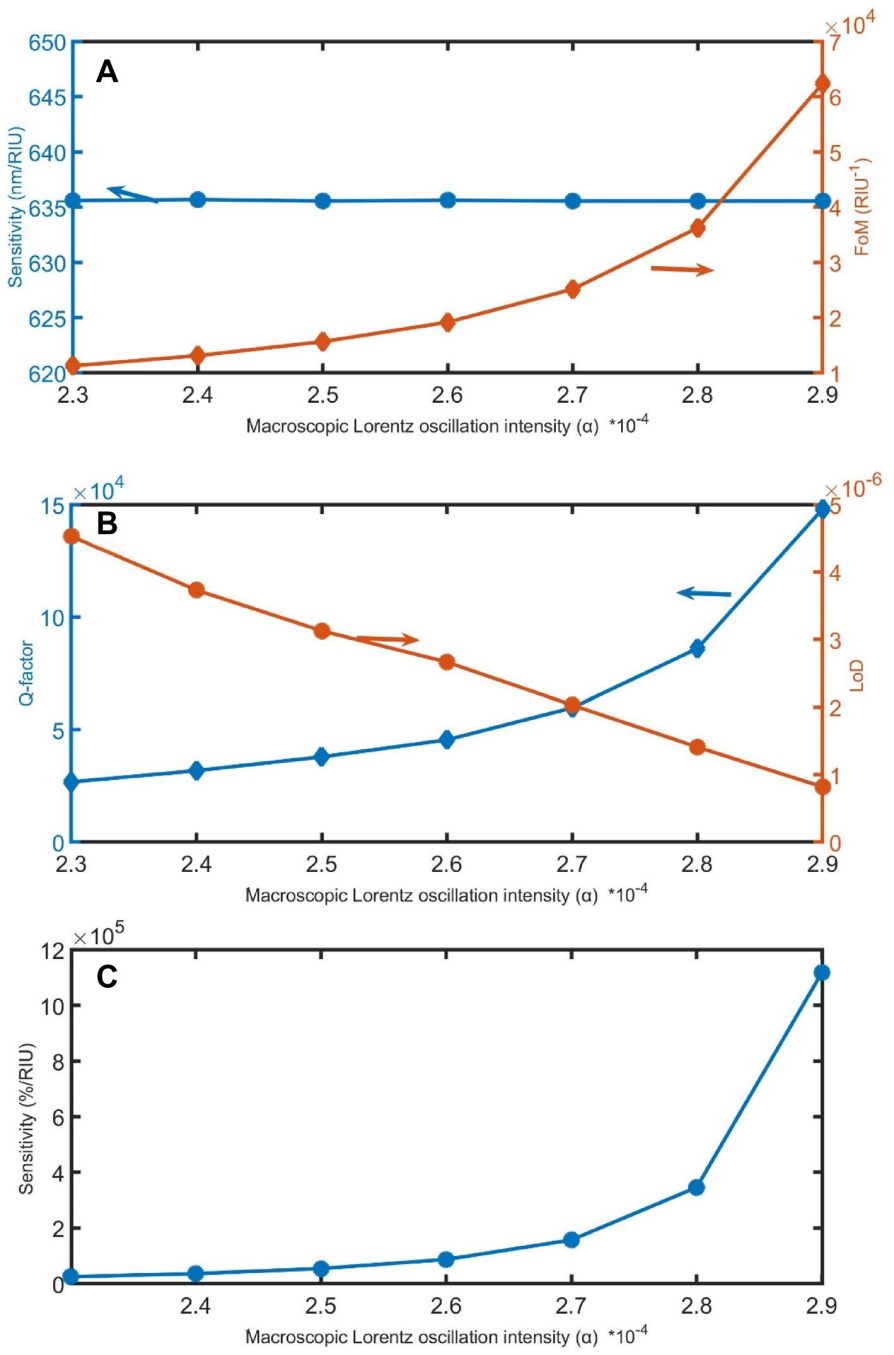
The impact of α on (**A**) sensitivity in nm/RIU and FoM, (**B**) Q and LoD, and (**C**) sensitivity in %/RIU.

As shown in Fig. 5A–C, when the macroscopic Lorentz oscillation intensity of both loss and gain layers increases from 2.3 × 10^–4^ to 2.9 × 10^–4^, the sensitivity in nm/RIU unit increase becomes linear and near to zero, FoM gradually increases from 11,277 to 62,364 RIU^−1^, Q gradually increases from 26,762 to 148,012, LoD linearly decreases from 5 × 10^–6^ to 8 × 10^–7^, and the increase in the sensitivity in %/RIU unit value strongly increases from 25,461 to 1,117,565 %/RIU.

**Figure 6:**
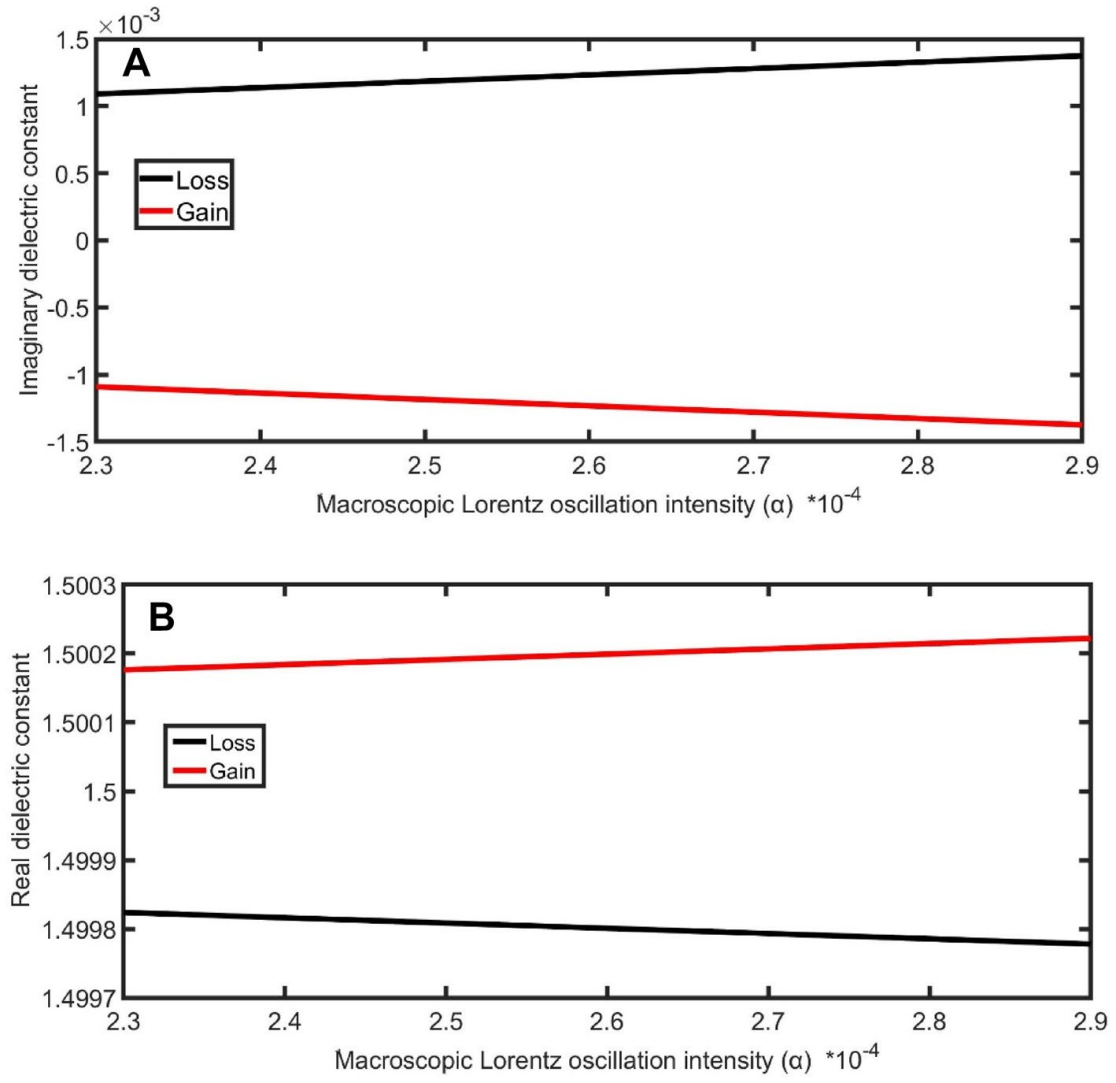
The impact of α on (**A**) imaginary dielectric constant, (**B**) real dielectric constant at 1525 nm as a function of macroscopic Lorentz oscillation intensity.

The real and imaginary dielectric constant of gain and loss layers are drawn and studied to analyze the impact of macroscopic Lorentz oscillation intensity on the magnification of transmittance. As clear in Fig. 6A,B, by increasing the macroscopic Lorentz oscillation intensity from 2.3 × 10^–4^ to 2.9 × 10^–4^, the gap between the real and imaginary part of the dielectric constants of loss and gain layers increases. As a result, the magnification of transmittance increases with the increase of the macroscopic Lorentz oscillation intensity and recorded the highest value of 1,117,565%/RIU at 2.9 × 10^–4^.

**Figure 7:**
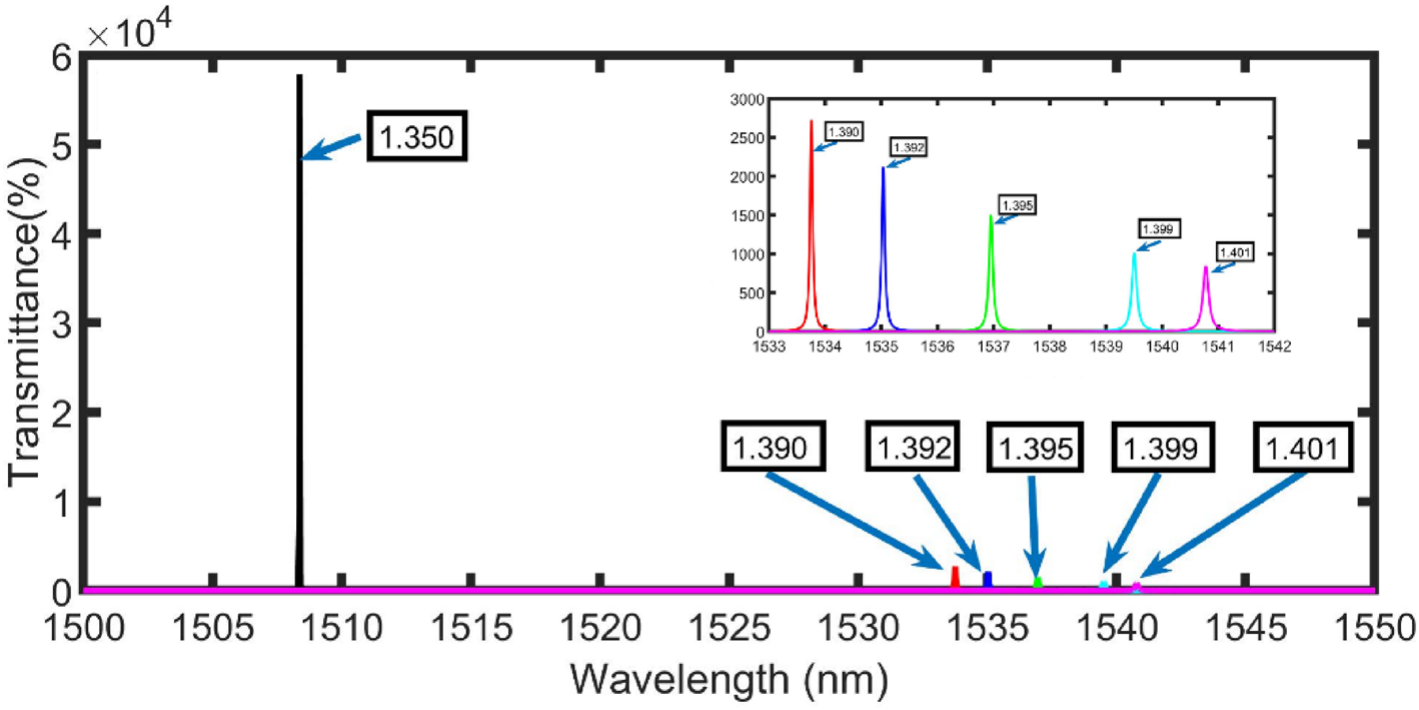
The transmittance of the proposed configuration at the selected conditions. The inset shows a zoom of the cancer cell peaks.

The transmittances of the proposed configuration at the selected conditions are calculated at different values of refractive indices from 1.350 to 1.390, 1.392, 1.395, 1.399, and 1.401 as clear in Fig. 7. The magnified resonant peak is shifted from 1508.37 nm to the right side at 1533.76, 1535.04, 1536.95, 1539.5, and 1540.78 nm as clear in Fig. 7 (inset figure). Besides, the transmittance of the magnified resonant peak is decreased from 57,843% (1.350) to 2726% (1.390), 2117% (1.392), 1502% (1.395), 1011% (1.399), and 847% (1.401).

**Figure 8:**
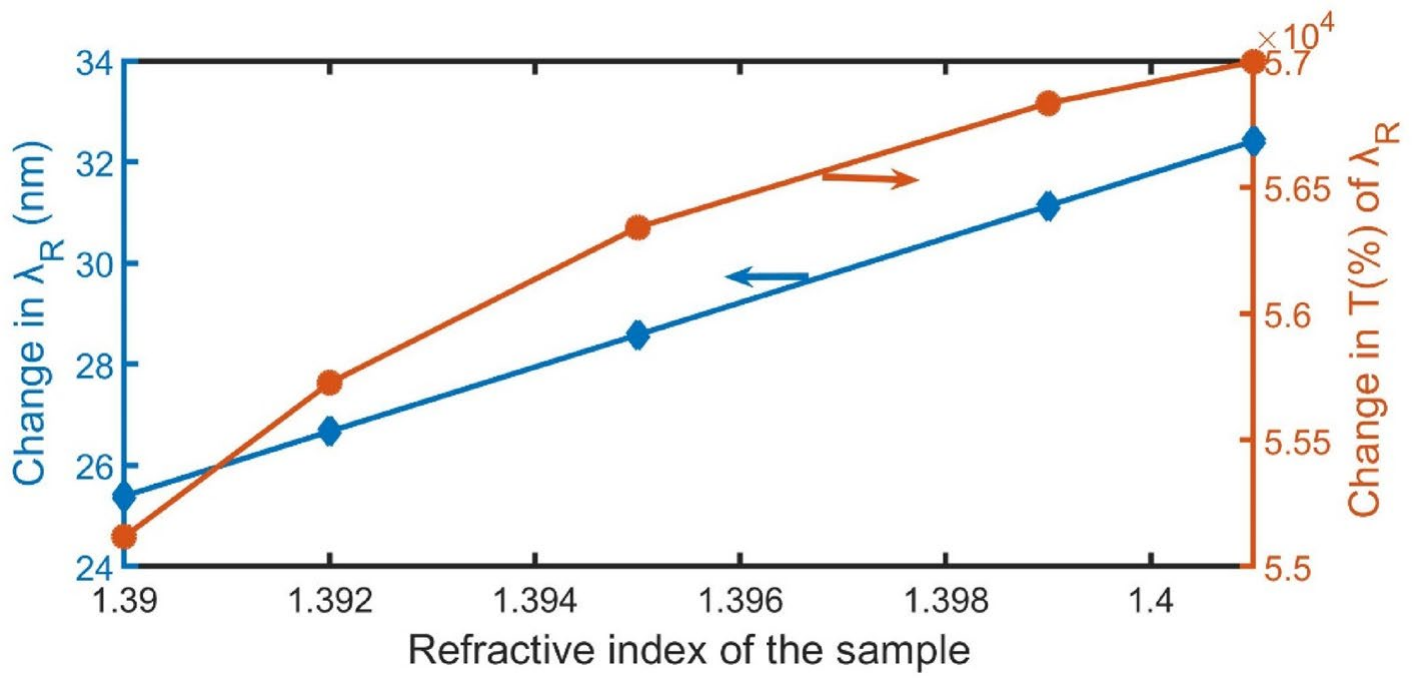
The position shift of the resonant peak and its transmittance versus the change in the refractive indices of samples.

The fluctuation of the magnified peak shift with the refractive index of the sample is seen in Fig. 8. As can be seen, the relationship between the magnified peak wavelength shift with the refractive index of the sample is linear, and not significantly linear for the change in transmittance intensity. Table. 1 summarizes the advantages of the suggested device compared to other recent works in terms of sensitivity, FoM, and Q-factor. The sensitivity, FoM, and Q-factor of our sensor are better than other studies in references^43–45^. The FoM in^46^ is better than ours, but our study outperformed it in the other parameters. In addition, our study outperformed all of them in the magnification of resonant peaks which makes it very distinguished. We expect that the proposed sensor has a lot of potential in the realm of optical devices.

**Table 1:** Comparative study (NC = not calculated).

Reference	S(nm/RIU)	FoM(/RIU)	Q-factor	Refractive index range	configuration
^43^, 2021	290	1074	2271	1.35 : 1.399	(MgF_2_/ZnSe)^N^/(graphene)/(defect)/(graphene)/(MgF_2_/ZnSe)^N^
^44^, 2021	344	NC	9138	1.35 : 1.41	(ZnSe/ZnS)^N^/(defect)/ (ZnSe/ZnS)^N^
^46^, 2021	273	90,000	10,000	1.00025: 1.0004	[prism/Au/air cavity/(TiO_2_ /SiO_2_)^10^]
^45^, 2022	81	580	4586	1.332: 1.6235	(ZnSe/Nb_2_O_5_/BK7)^N/2^/(defect)/ (ZnSe/Nb_2_O_5_/BK7)^N/2^
This work	635	62,364	148,012	1.35: 1.401	(loss/PSi/gain)^N^/sample/(loss/PSi/gain)^N^

### Fabrication tolerance

**Figure 9:**
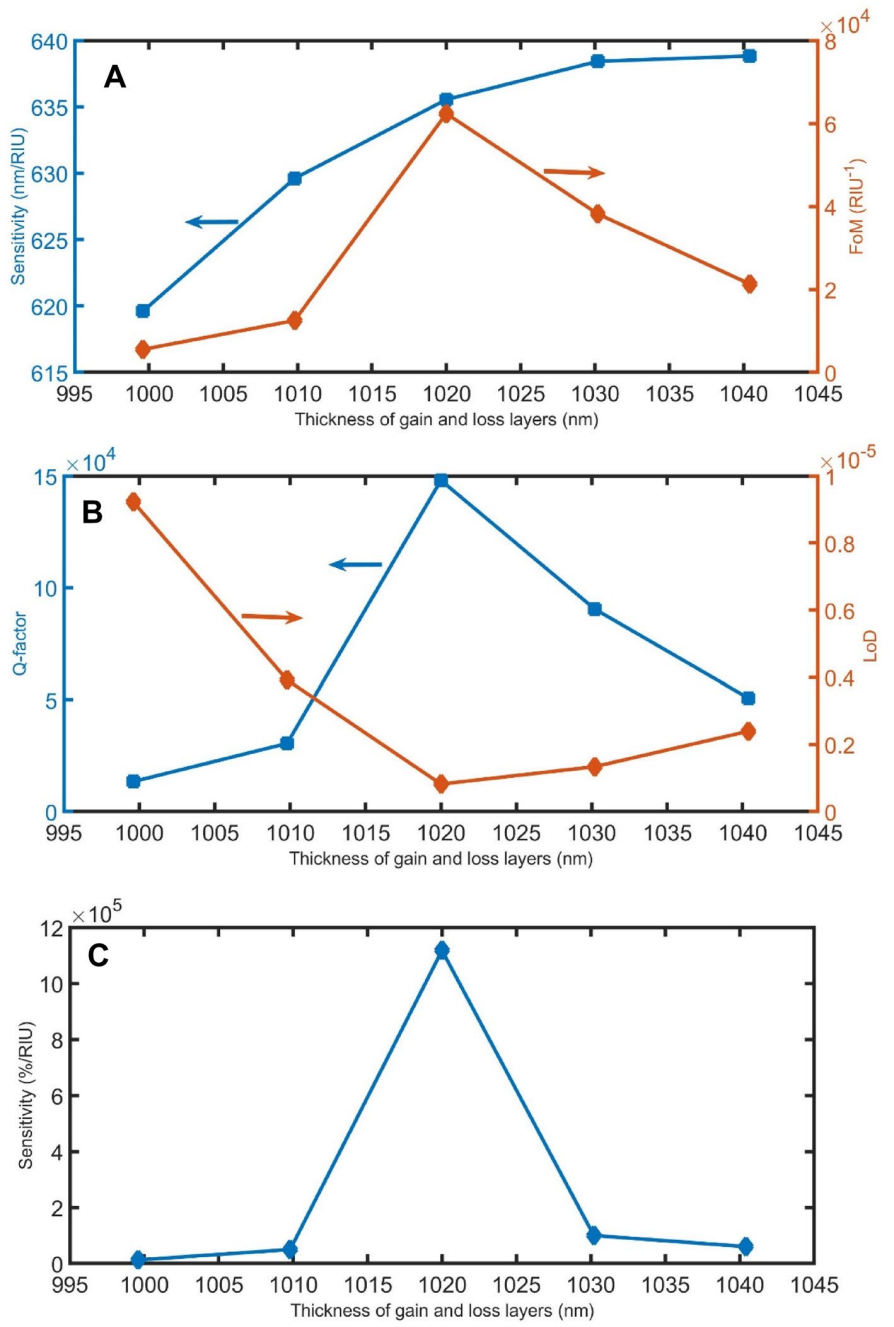
The (**A**) sensitivity in nm/RIU and FoM, (**B**) Q-factor and LoD, and (**C**) sensitivity in %/RIU, with varying the thickness of the gain and loss layer.

To ensure that the proposed sensor has good stability, the effects of fabrication tolerance^47^ of most parameters in the range of ± 2% except incident angle (+ 10%) on the sensing performance are studied. The impact of the thickness of the gain and loss layers on the performance of the sensor is the first fabrication tolerance to be addressed. As it is difficult to adjust the thickness of the gain and loss layers at an exact thickness of 1020 nm, we will study the performance of the sensor in the range of 1020 nm ± 2%. When the thickness of the gain and loss layers change from 999.6 to 1040.4 nm as in Fig. 9, the resonant peaks are slightly shifted from 1499.6 to 1516.9 nm for n = 1.35, and from 1531.2 to 1549.5 nm for n = 1.401, according to Bragg–Snell’s law. Even though this shift in resonant peaks, the difference between resonant peaks at each thickness ($\Delta {\uplambda }_{R}$) seems to be constant (slightly changes from 31.6 to 32.58 nm). As a result, the sensitivity in the nm/RIU unit slightly increased from 619.6 to 638.8 nm. Besides, FoM is changed between 0.5 × 10^4^ and 6 × 10^4^ /RIU, as clear in Fig. 9A. For Q-factor, it is changed between 1.3 × 10^4^ and 14 × 10^4^. LoD also is fluctuated between 0.08 × 10^–5^ and 0.9 × 10^–5^, as clear in Fig. 9B. In Fig. 9C, the sensitivity in the %/RIU unit is changed between 0.1 × 10^5^ and 11.1 × 10^5^.

**Figure 10:**
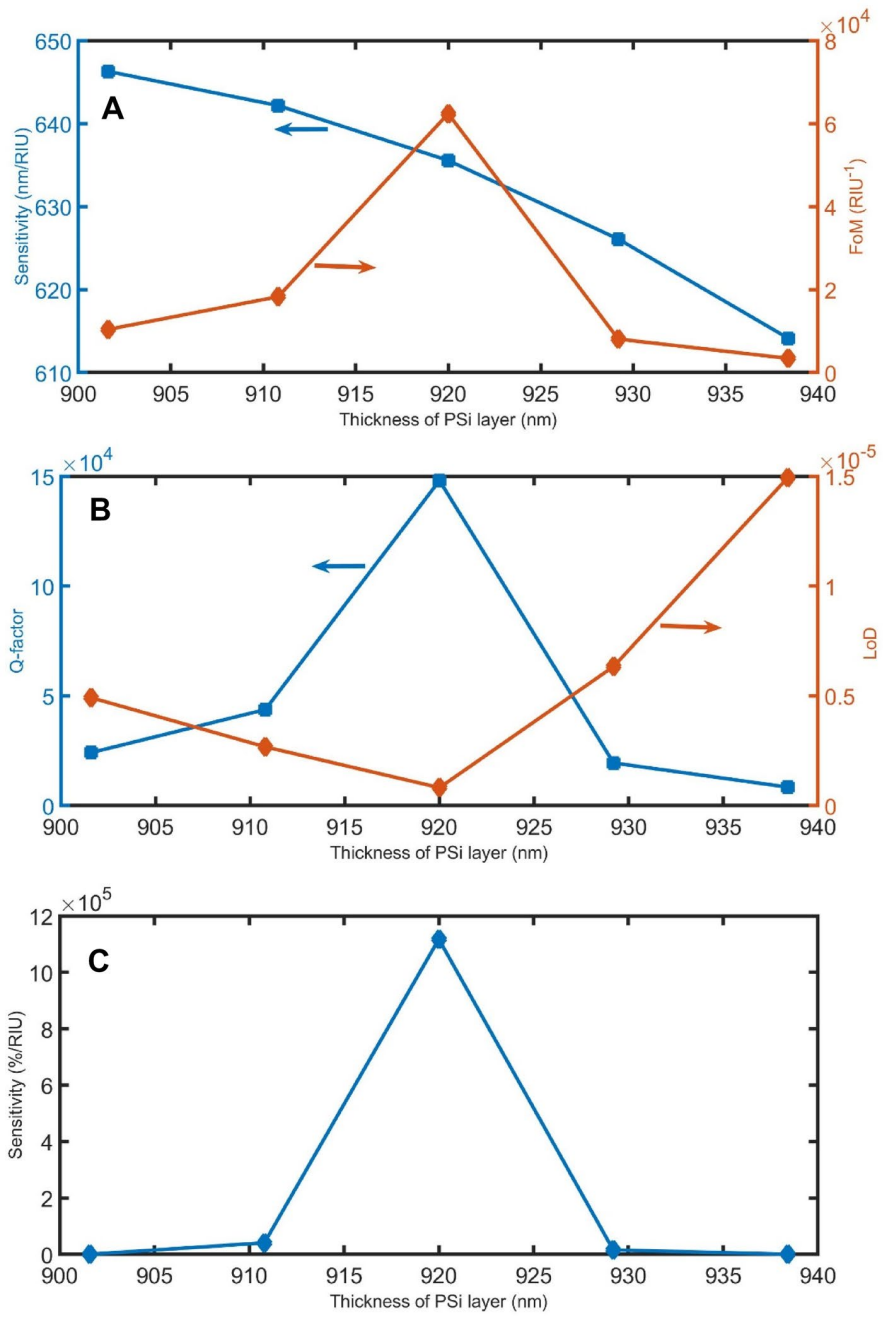
The (**A**) sensitivity in nm/RIU and FoM, (**B**) Q-factor and LoD, and (**C**) sensitivity in %/RIU, with varying the thickness of the PSi layer.

As clear in Fig. 10, the effect of fabrication tolerance of the PSi layer thickness on the sensor’s performance will be studied in the range of 920 nm ± 2%. If the thickness of the PSi layer changes from 901.6 to 938.4 nm, the resonant peaks are slightly shifted from 1502.0 to 1515.5 nm for n = 1.35, and from 1535.0 to 1546.8 nm for n = 1.401, according to Bragg–Snell’s law. Also, the $\Delta {\uplambda }_{R}$ slightly decreased from 33.0 to 31.32 nm). As a result, the sensitivity in the nm/RIU unit slightly decreased from 646.3 to 614.1 nm/RIU. FoM is fluctuated between 0.3 × 10^4^ /RIU and 6 × 10^4^ /RIU, as clear in Fig. 10A. In the case of the Q-factor, it changed between 2.4 × 10^4^ and 14.8 × 10^4^. LoD is varied between 0.08 × 10^–5^ and 1.5 × 10^–5^, as clear in Fig. 10B. The sensitivity in the %/RIU unit is changed between 0.003 × 10^5^%/RIU and 11.2 × 10^5^%/RIU, as clear in Fig. 10C.

**Figure 11:**
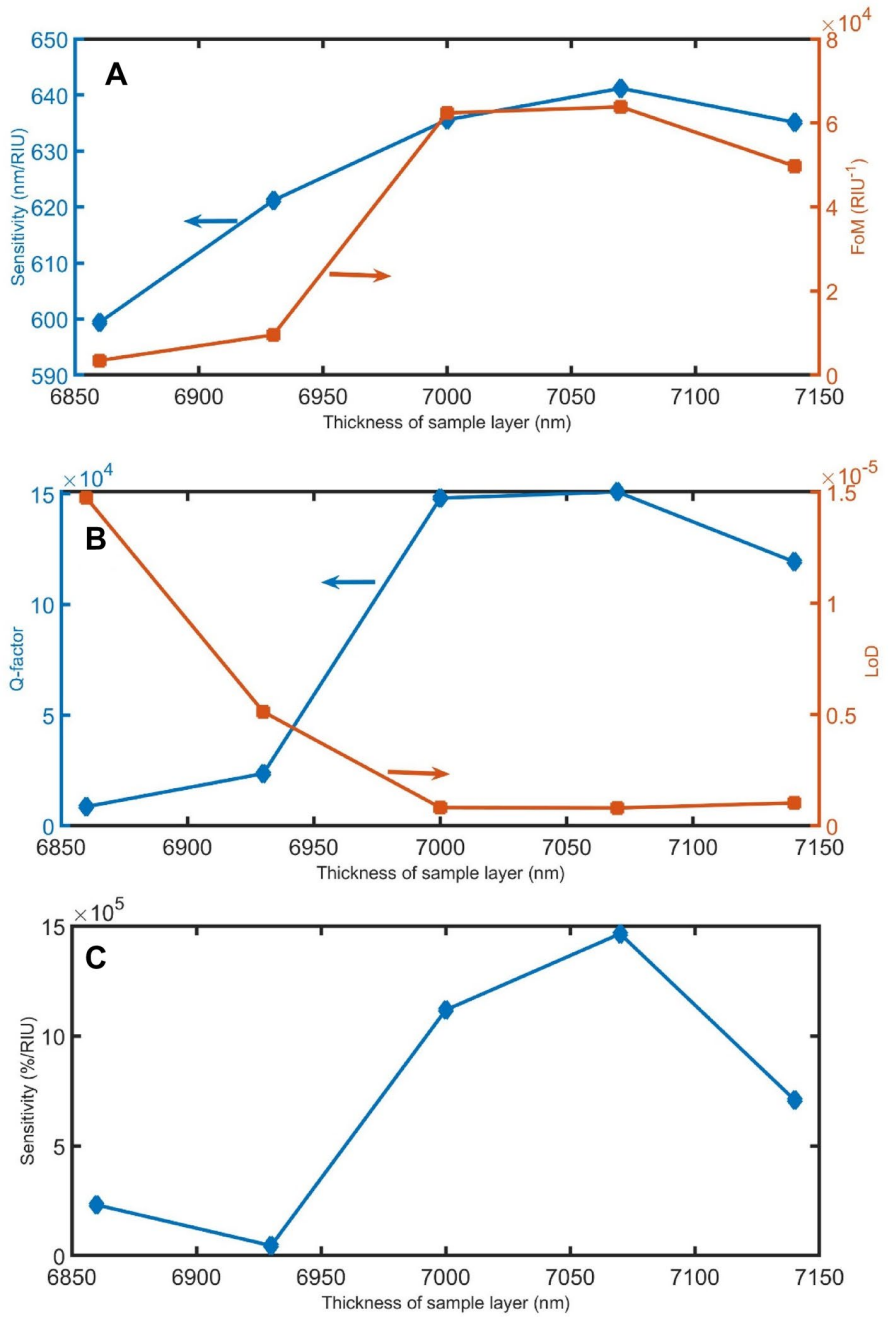
The (**A**) sensitivity in nm/RIU and FoM, (**B**) Q-factor and LoD, and (**C**) sensitivity in %/RIU, with varying d_sample_.

**Figure 12:**
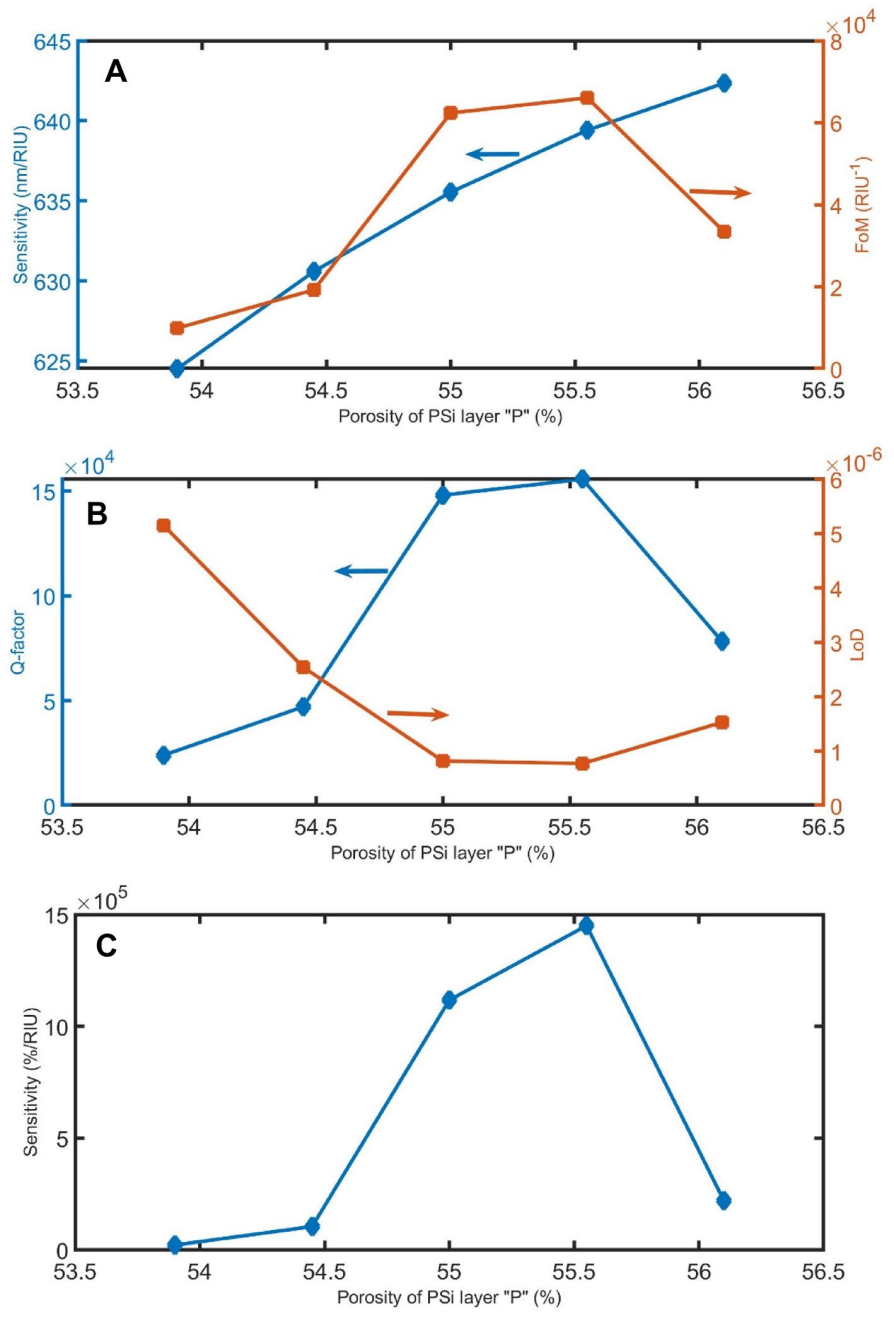
The (**A**) sensitivity in nm/RIU and FoM, (**B**) Q-factor and LoD, and (**C**) sensitivity in %/RIU, with varying the porosity of PSi.

**Figure 13:**
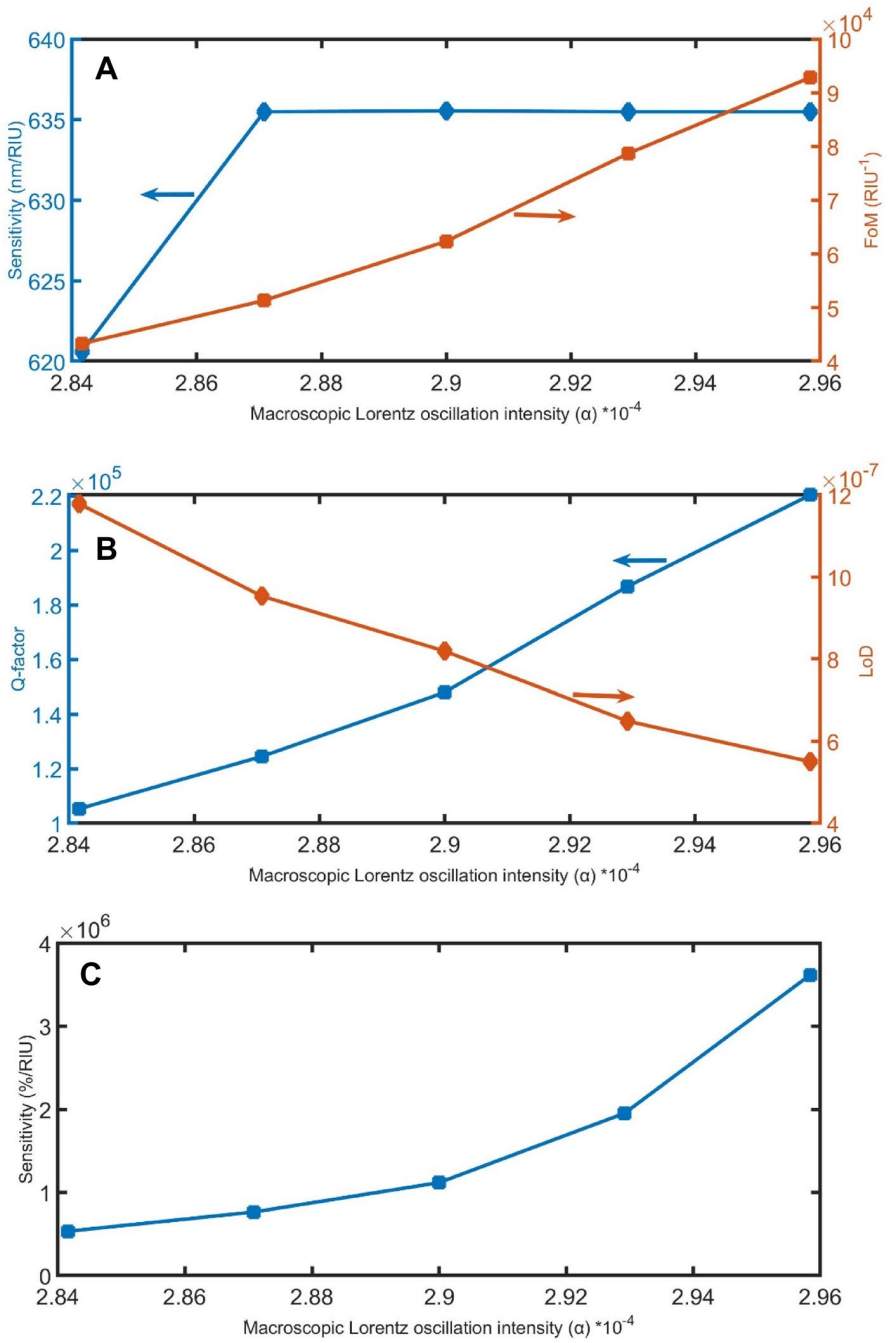
The (**A**) sensitivity in nm/RIU and FoM, (**B**) Q-factor and LoD, and (**C**) sensitivity in %/RIU, with varying the α.

**Figure 14:**
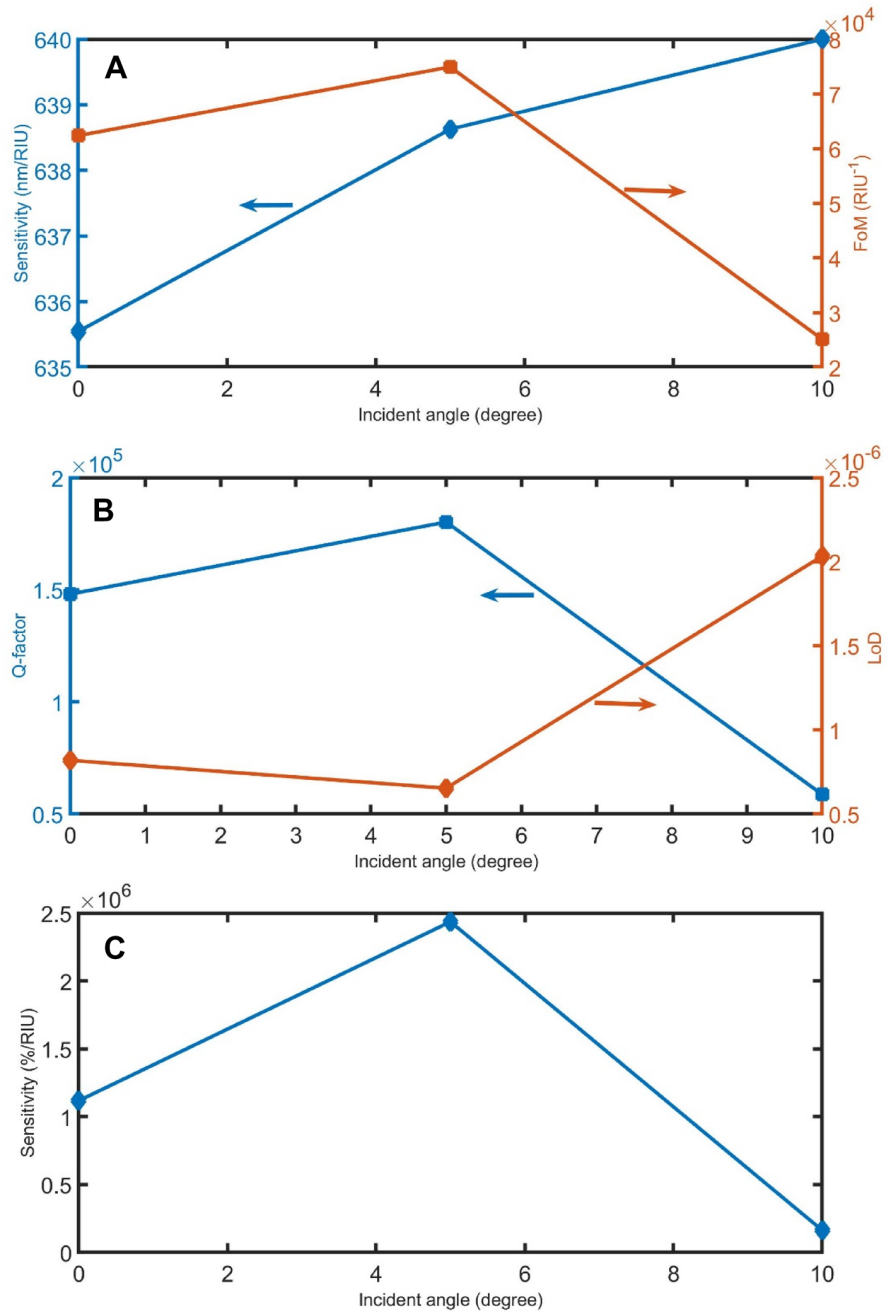
The (**A**) sensitivity in nm/RIU and FoM, (**B**) Q-factor and LoD, and (**C**) sensitivity in %/RIU, with varying the incident angle.

Figures 1–14 demonstrate the effect of the parameters d_sample_, P, α, and ${\theta }_{0}$ on the performance of the sensor as other manufacturing tolerances. As clear in Figs. 11A, 12A, 13A, and 14A, the sensitivity in the nm/RIU unit is less affected by the manufacturing tolerances of these parameters, indicating that the proposed device has acceptable stability within the range of ± 2% except incident angle (+ 10%). However, as the fabrication tolerances of d_sample_, P, α, and ${\theta }_{0}$ increase, the FoMs change from 0.3 × 10^4^ to 6.2 × 10^4^ /RIU, from 0.1 × 10^4^ to 6.6 × 10^4^ /RIU, from 4.3 × 10^4^ to 9.3 × 10^4^ /RIU, and from 2.5 × 10^4^ to 7.5 × 10^4^ /RIU, respectively. As clear in, Figs. 11B,C, 12B,C, 13B,C, and 14B,C the Q-factor LoD and sensitivity in the %/RIU unit are varied by changing the fabrication tolerances of these parameters.

Finally, the proposed sensor records acceptable stability for sensitivity in the nm/RIU unit. Even though FoM, Q-factor, LoD, and sensitivity in the %/RIU unit have shown that their values will be slightly affected by fabrication tolerances, the proposed sensor on all conditions records a very high performance relative to other devices as clear in Table 1. This variability in the performance with fabrication tolerances doesn’t mean that the proposed sensor is not stable. After fabrication, it can be calibrated to determine the real relation between the peak position and its refractive index. According to our knowledge, the main advantage of this sensor is that the resonant peaks are magnified for all mentioned conditions. Besides, we are still hoping new experimental techniques for depositing multilayers with limited tolerances (0%) to be discovered in the near future.

In conclusion, a refractive index sensor with magnified resonant peaks and high sensitivity was proposed. TMM-based simulations demonstrate that the proposed sensor can detect refractive index with high sensitivity and linearity. Besides, results show that the position and transmittance sensitivities are 635 nm/RIU and 1,000,000%/RIU, respectively. Because of its magnification, wide sensing range, high sensitivity, and ease of manufacture, the suggested sensor is thought to have a lot of potential in detection applications.

## Data Availability

Requests for materials or code should be addressed to Zaky A. Zaky.
